# Two-Photon Vision in Age-Related Macular Degeneration: A Translational Study

**DOI:** 10.3390/diagnostics12030760

**Published:** 2022-03-21

**Authors:** Grzegorz Łabuz, Agnieszka Zielińska, Lucy J. Kessler, Asu Rayamajhi, Katarzyna Komar, Ramin Khoramnia, Gerd U. Auffarth

**Affiliations:** 1 The David J Apple Center for Vision Research, Department of Ophthalmology, University Hospital Heidelberg, Im Neuenheimer Feld 400, 69120 Heidelberg, Germany; lucyjoanne.kessler@med.uni-heidelberg.de (L.J.K.); asu.rayamajhi@kit.edu (A.R.); ramin.khoramnia@med.uni-heidelberg.de (R.K.); gerd.auffarth@med.uni-heidelberg.de (G.U.A.); 2 Institute of Physics, Faculty of Physics, Astronomy and Informatics, Nicolaus Copernicus University in Toruń, Grudziądzka 5, 87-100 Toruń, Poland; azielinska@fizyka.umk.pl (A.Z.); kkomar@fizyka.umk.pl (K.K.); 3 Karlsruhe Institut für Technology (KIT), Light Technology Institute (LTI), Engesserstraße 13, Bldg. 30.34, 76131 Karlsruhe, Germany; 4 International Centre for Translational Eye Research, Skierniewicka 10A, 01-230 Warsaw, Poland; 5 Department of Physical Chemistry of Biological Systems, Institute of Physical Chemistry, Polish Academy of Sciences, Kasprzaka 44/52, 01-224 Warsaw, Poland

**Keywords:** two-photon vision, AMD, normal aging, microperimetry, retinal sensitivity

## Abstract

The recently introduced term “two-photon vision” relates to the visual perception resulting from a simultaneous absorption of two photons by photoreceptors. In this study, we determined two-photon retinal sensitivity in age-related macular degeneration (AMD) and compared it that in normal aging. Microperimetry was performed with visible (white) light and infrared (IR) light, which was perceived as green in the two-photon stimulation. In total, 45 subjects were included with one (better) eye studied. Furthermore, best-corrected visual acuity (VA) and ocular straylight were assessed. AMD resulted in decreased median (interquartile range) logMAR VA, i.e., 0.15 (0.05; 0.24), which in normal eyes was −0.02 (−0.06; 0.02). The two groups showed comparable straylight levels. Sensitivity to IR light was significantly lower in the AMD group (*p* < 0.001): 8.3 (7.4, 9.3) dB than in controls 10.7 (9.7, 11.2) dB. AMD also significantly affected visible light sensitivity (*p* < 0.001): 14.0 (11.0; 15.5) dB vs. 18.0 (16.3; 18.9) dB. Notably, the two-photon approach yielded a lower data spread. In conclusion, AMD considerably impairs retinal sensitivity measured in the single- and two-photon realm. However, two-photon-vision microperimetry may improve the testing accuracy and offer an additional diagnostic parameter (beyond VA measurements) for retinal function assessment.

## 1. Introduction

Standard visual perception results from a single photon response, delimited in spectral sensitivity of 380 to 700 nm [1]. Recently, a new concept of visual perception has been proposed, which involves the simultaneous absorption of two photons instead of one [2,3]. Pulsed infrared (IR) laser can trigger two photon absorption and produce color sensation corresponding to half of the wavelength of the stimulating beam (e.g., blue or green) [2,3,4] despite that high-power IR emitters are typically seen as red or colorless in single-photon perception [5]. The relationship between the brightness and the power of the stimulating beam is quadratic for the two-photon perceived stimuli. This attribute of two-photon vision may be advantageous, especially for the accuracy of visual threshold determination. A recent laboratory study demonstrated that the spread of the psychometric function for detecting two-photon stimuli was narrower (by a factor of 2) than for single-photon testing [3]. The repeatability of two-photon visual threshold measurements was also improved compared to standard (visible-light) stimuli [3]. Although both types of photoreceptors mediate two-photon vision [2], studies of dark adaptation curves measured at 520 nm and 1040 nm have shown that the sensitivity difference between the two photoreceptors is substantially smaller than for a single-photon process [3]. The pupil response is also significantly smaller for two-photon versus standard targets having the same color and brightness [6]. Since the two-photon visibility threshold is a new parameter to assess retinal function, its clinical application has only recently been sought [4,7,8].

In an initial study, we established the normative level of two-photon IR light sensitivity in adults aged 20 to 70 [4]. We subsequently demonstrated that diabetic retinopathy patients have significantly decreased retinal sensitivity to IR light compared to their age-matched controls [7]. A potential implementation of this new technology in the management of retinal diseases was proposed [4,7]. Additionally, in a recent analysis, the two-photon assessment has proven supportive in the detection of glaucomatous neuropathy [8]. We found a significant correlation between optical coherence tomography (OCT) retinal biomarkers and IR light sensitivity, suggesting another application area.

Age-related macular degeneration (AMD) is a neurodegenerative disease affecting the macula, and it can result in severe loss of central vision [9,10]. Given the progressive, irreversible damage to photoreceptors and their subsequent atrophy, one may expect that the two-photon absorption is compromised in equal measure to the damage made by AMD to normal vision. This degenerative disease also affects other layers of the retina, e.g., retinal pigment epithelium (RPE), Bruch’s membrane, and choriocapillaris [10]. A higher level of lipofuscin and decreased concentration of melanosomes have been reported as potential biomarkers of AMD [11]. An increased number of confluent extracellular drusen, geographic atrophy (GA) of the RPE resulting in the loss of photoreceptors, and retinal pigment epithelial detachments are typical morphological features of nonexudative AMD. The exudative form is characterized by subretinal neovascularization due to abnormal growth of retinal blood vessels associated with the presence of subretinal fluid [10].

Although such changes are often seen in standard fundoscopy, routinely performed by clinicians, the diagnosis and management of AMD patients are commonly supported by imaging techniques [9]. Fundus photography has been particularly useful in disease-progression monitoring by providing a baseline image for subsequent appointments. The inclusion of spectral filters, e.g., autofluorescence imaging or fundus angiography, offers additional tools for improved detection of eye pathology [9]. The introduction of OCT proved an important milestone in advancing AMD diagnosis as OCT increased the depth of examination by allowing a non-invasive assessment of the retinal anatomical structure. These advances in ophthalmology extended our understanding of the pathomechanism of various retinal disorders and expanded clinicians’ range of diagnostic tools. OCT proved valuable in diagnosing exudative AMD because with its high spatial resolution, even small subretinal fluid pockets or choroidal neovascularization can be detected [9].

Microperimetry, which involves retinal sensitivity testing coupled with fundus imaging, has added a functional component to the morphological assessment [12]. As opposed to standard (static) perimetry, microperimetry covers a narrow portion of the visual field, typically limited to the macula. Following a microperimetric examination, an exact retinal location of test points and measured sensitivity are superimposed over the patient’s fundus recording, which establishes a link between structure and function [12]. Microperimetry has been applied successfully in studying the functional effects of AMD and the efficacy of treatment methods beyond standard visual acuity (VA) [12,13,14,15]. Fundus-driven perimetry has primarily been performed in a single-photon realm. However, the transition to two-photon retinal sensitivity may prove advantageous given its higher tolerance to eye turbidity and the nonlinearity of two-photon vision, which may yield more accurate testing [3].

In this study, we measured retinal sensitivity in AMD patients with standard visible-light microperimetry and a two-photon approach and compared their performance against a control group to evaluate the application of two-photon technology in the assessment of AMD.

## 2. Materials and Methods

### 2.1. Subject Selection

Study participants were recruited from the outpatient department of Heidelberg University Eye Clinic. This study adhered to the provisions of the Declaration of Helsinki and was approved by the Ethics Committee of Medical Faculty Heidelberg of Heidelberg University. All participants gave their written informed consent following a detailed description of the study protocol.

A clinical classification proposed by Ferris et al. was applied to grade the severity of AMD (exudative and nonexudative) [16]. Patients with medium drusen with a size range from 63 µm to 125 µm had an early form (grade I). Eyes with larger or confluent drusen (>125 µm) irrespective of pigmentary abnormalities were classified as grade II (intermediate AMD), and neovascularization or geographical atrophy resulted in grade III (late or advanced AMD). Patients with other ocular pathology (e.g., glaucoma) or systemic diseases (e.g., diabetes) were excluded. The control group subjects had (a) drusen < 63 µm, which is considered to be normal aging [16], and (b) Snellen visual acuity (VA) equal to or better than 0.8 (0.1 logMAR). Although we established no minimum VA limit, the better eye was always selected for examination and statistical analysis. An absolute spherical component of the refractive error had to be <4 D and astigmatism < 1.5 D due to a limited refractive error correction range implemented in the study setup.

### 2.2. Study Procedures

**Figure 1:**
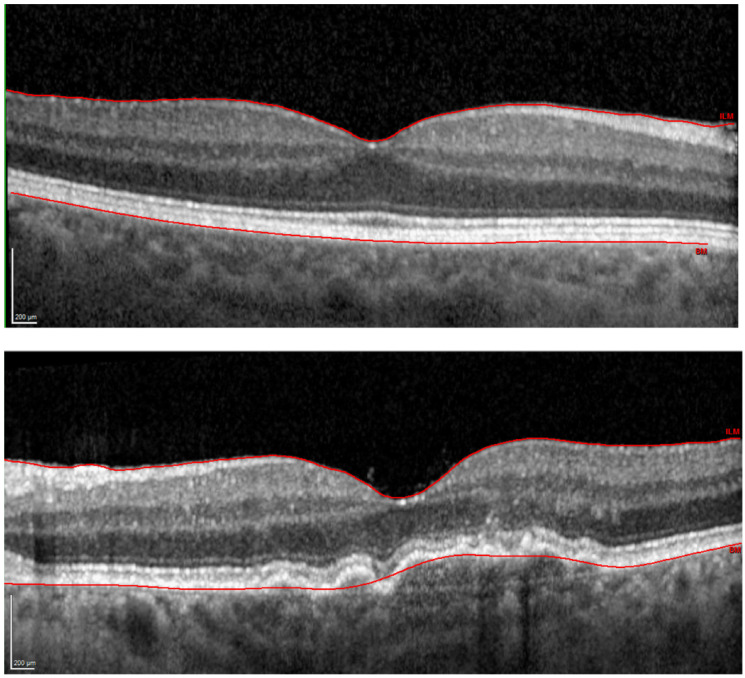
Optical coherence tomography images of a representative control (upper panel) and disease (lower panel) case. The solid red line indicates the automatic retinal layer segmentation, which defines the retinal thickness.

Following successful enrollment based on subjects’ medical history, monocular best-corrected VA (BCVA) was assessed using an Early Treatment Diabetic Retinopathy Study (ETDRS) chart placed at 4 m. The logarithm of the straylight parameter, i.e., log(s), was measured using a C-Quant device (Oculus GmbH, Wetzlar, Germany) to quantify ocular turbidity [17]. Next, OCT with Spectralis (Heidelberg Engineering GmbH, Heidelberg, Germany) was performed after pupil dilation with 5 mg/mL tropicamide (Pharma Stulln GmbH, Stulln, Germany). A 30° scan was taken for macula assessment and the retinal thickness, which is the distance separating the internal limiting membrane and the outer surface of the RPE (Figure 1), was exported using built-in software. A comprehensive examination of the anterior segment and the fundus was conducted with a slit lamp to render patients’ eligibility for the study and grade AMD severity.

Following 5-min dark adaptation, retinal sensitivity was measured in one (better) eye. If both eyes had equal VA, the selection was randomized with randomization software (https://www.random.org/, accessed on 20 February 2022). Before beginning the test, we gave an extensive explanation to each subject about the procedure, including practice trials.

**Figure 2:**
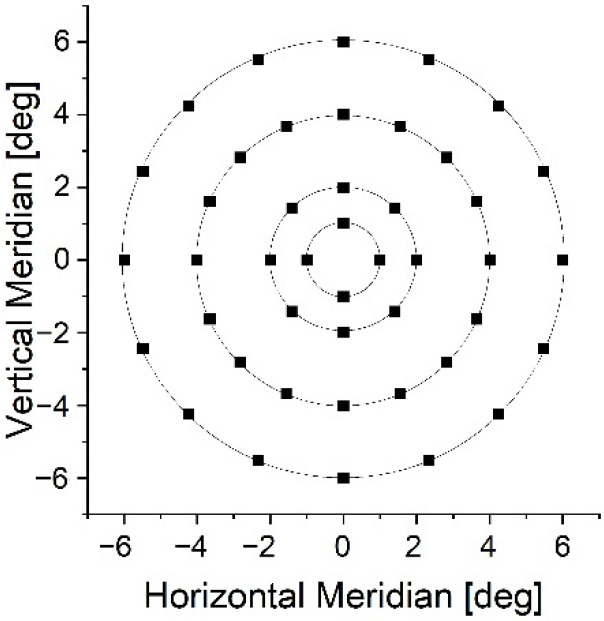
The distribution of retinal loci for sensitivity testing.

White light sensitivity was assessed using an MP1 microperimeter (Nidek Technologies Srl, Albignasego, Italy) [8,13,14,15], which features an eye tracker and fundus camera that automatically records a 45°-field-of-view image of the retina. We used a customized test grid consisting of 44 points and spanning 6° around the fovea (Figure 2). Goldmann III stimuli were projected by a liquid-crystal display featuring a 1.27 cd/m^2^ luminance background. For testing, a red, 0.5° fixation point, a 4-2-1 strategy, and a 200 ms stimulus duration were selected.

IR light sensitivity was assessed using a customized optical system. A detailed description of the setup can be found elsewhere [4,7,8]. In brief, a femtosecond laser (HighQ-2, Spectra-Physics, Milpitas, CA, USA) and a set of galvo scanners controlled by customized software direct ultrashort pulses of 1045-nm light at the retina. Note that the laser power was well below safety limits stipulated by ANSI Z136.1-2014 and EN 60825-1:2014 requirements due to a controlled light loss in the optical path and a set of neutral-density filters. A trajectory of each projection was predefined and executed to consecutively draw a circular (not-filled) pattern in each position. Measurement settings were adjusted to mimic MP1 conditions in terms of the stimulus size and presentation time, as well as the background luminance. A monochromatic (630 nm) light-emitting diode with a diameter of 0.1° served as a fixation target. Although the same grid point was applied for IR light testing (Figure 2), a standard staircase procedure with the stimulus intensity increasing monotonously until it became noticeable replaced the 4-2-1 strategy [18]. Two measurements per retinal loci were taken in a randomized order. The subject used a computer mouse button to indicate the presence of the stimulus. IR light sensitivity examination time (approx. 13 min) in healthy subjects was longer than with the MP1 device (approx. 7 min). Although our setup does not feature eye tracking, careful monitoring of patient gaze stability was performed in real-time using fundus imaging, produced by a scanning laser ophthalmoscope (SLO) system operating at 880 nm, and a pupil preview camera with a 950-nm illumination ring.

The two-photon excitation setup provides a broad dynamic range from 0 dB (400 µW) to 26 dB (1 µW), which is expressed in radiometric units, given the use of IR light. The MP1 uses white light with a minimum and maximum luminance (photometric units) of 1.27 cd/m^2^ (20 dB) and 127 cd/m^2^ (0 dB), respectively.

### 2.3. Statistical Analysis

**Figure 3:**
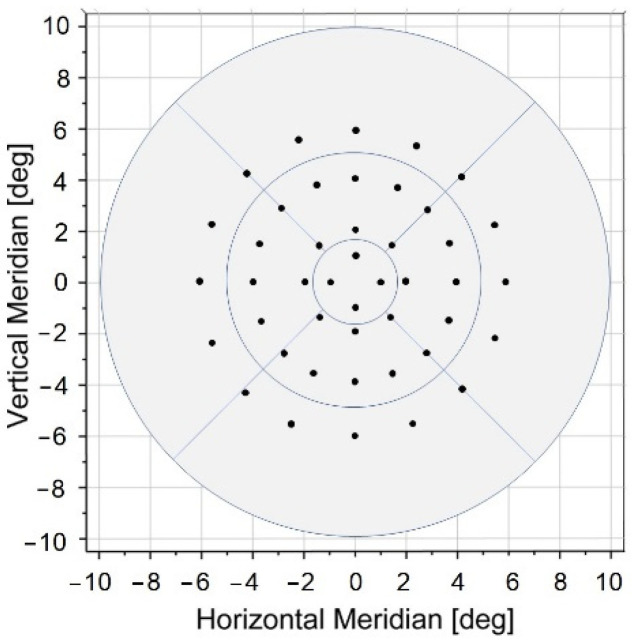
Grid point distribution superimposed over a macular thickness map with radial sector borders corresponding to 1 mm, 3 mm, and 6 mm ETDRS.

Non-parametric methods were applied with descriptive statistics given as a median (interquartile range; IQR) due to the skewness of MP1 retinal sensitivity results. Mann–Whitney U test was performed to assess the significance of (pooled) sensitivity differences between the disease and control groups as well as age. A *p*-value of less than 0.05 was set to indicate a statistically significant difference. Left eye visual field results were right eye transposed. A pointwise comparison of microperimetry data was performed with triangular interpolation to simulate an XYZ visual field representation. In addition, a radial stratification of points having the same angular range (Figure 2) was applied, which resulted in the formation of four subgroups with a radius from the fovea of 1°, 2°, 4°, and 6°. Finally, Spearman’s rank correlation coefficient (denoted as ρ) was used to test the usefulness of the central subfield thickness in predicting retinal sensitivity changes. To this end, we selected 4 loci occupying the center of the test grid (Figure 3) and calculated their mean sensitivity. The resulting values were then correlated with the retinal thickness deviation from a normal (287.5 µm) level found in the current study. We used OriginPro 2020 (OriginLab, Corporation, Northampton, MA, USA) for data analysis and visualization.

## 3. Results

The study population comprised 45 subjects: 23 in the AMD group and 22 controls. Of the 23 AMD eyes, five were classified as grade I, seven as grade II, and 11 as grade III. The median (IQR) age of the AMD subjects was 77.3 (72.2; 79.9) years and 71.6 (68.0; 77.9) years for the controls, which did not yield a statistically significant difference (*p* = 0.07). BCVA was compromised in the disease group compared to non-AMD patients, which was 0.15 (0.05; 0.24) logMAR vs. −0.02 (−0.06; 0.02) logMAR. Median spherical equivalent was 0.00 D in both groups. Similarly, the level of straylight was also comparable, as we found 1.17 (1.06; 1.45) log(s) in AMD and 1.18 (1.01; 1.29) log(s) in normal eyes.

**Figure 4:**
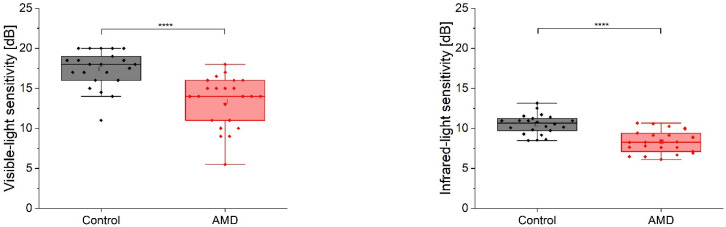
Two-group comparison for visible (left panel) and infrared light (right panel) sensitivity assessment. The box width indicates the interquartile range. The whiskers denote the fifth and 95th percentiles; the open squares refer to the mean value; and solid lines indicate the median level; points are individual data. **** *p* < 0.001.

**Figure 5:**
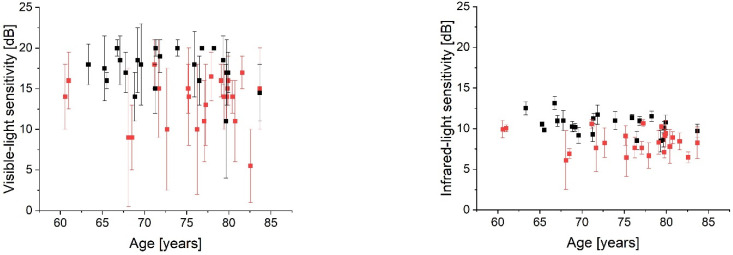
Retinal sensitivity to visible (left panel) and infrared light (right panel) as a function of age. The median value over 44 points assessed in each AMD (red) and control (black) subject was taken. Error bars = interquartile range.

The median sensitivity to IR light was 8.3 (7.2, 9.3) dB in the AMD cases and 10.7 (9.7, 11.2) dB in the normal population. The MP1 assessment showed 14.0 (11.0; 15.5) dB for disease and 18.0 (16.3; 18.9) dB for control subjects. Both comparisons (Figure 4) yielded statistically significant differences (*p* < 0.001). Figure 5 shows the age distribution of the (pooled) retinal sensitivity. The correlation coefficient (ρ) found in the control group was −0.35 for IR- and −0.11 for visible-light; however, it did not reach significance level (*p* > 0.05) for both approaches. An increased intrasubject variability (manifested by a higher IQR) can be observed in the MP1 results of both cohorts (Figure 5).

**Figure 6:**
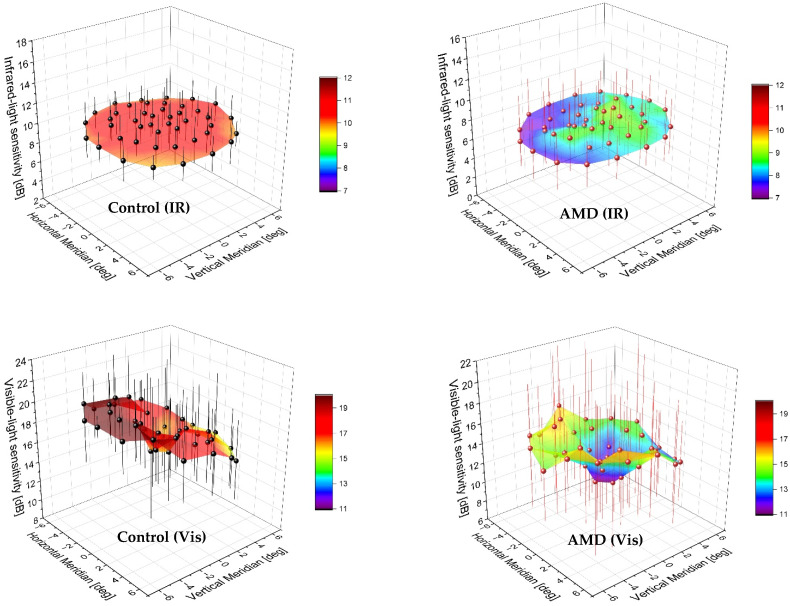
Retinal sensitivity maps of control (left panels) and AMD (right panels) participants compared using visible-light (Vis) microperimetry (upper panels) and two-photon mediated infrared light (IR) stimulation (lower panels). A triangular approach was applied to interpolate the area (colored gradient) between 44 measured loci (solid points). Control subjects’ results were marked in black; red marking indicates AMD patients. Error bars = interquartile range.

The distribution of sensitivity in each retinal locus is visualized in Figure 6. Both methods confirmed the debilitating effect of AMD on retinal sensitivity with a decreased distribution profile. The two-photon map of the controls produced a nearly uniform data spread with a slight increase in the center. Note that sensitivity was not tested at the fixation point.

The presence of AMD upsets this uniform distribution, showing a sensitivity peak shift and a valley starting near 1° and extending temporally to 6°. The MP1 measurements of the normal and AMD eyes yielded a depression in the center of the testing area. A tilt of the surface map indicated nonuniformity of retinal sensitivity results, which was less pronounced in AMD. Although the IQR recorded in the AMD cases was comparable between the two methods, the visible-light microperimetry appeared to exhibit higher intersubject variability in the control group.

**Figure 7:**
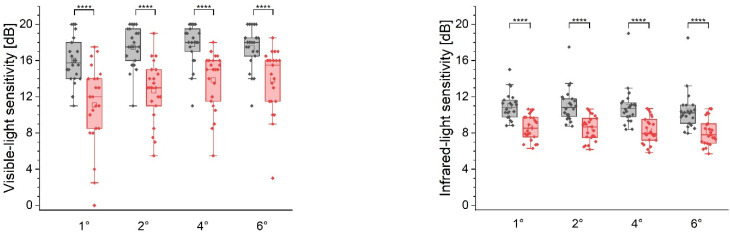
Box plots of the sensitivity results obtained along radially distributed retinal loci with a 1°, 2°, 4°, and 6° radius from the fovea. Note that the box width indicates the interquartile range. The whiskers denote the fifth and 95th percentiles; the outliers are marked as filled circles. The open squares refer to the mean value and solid lines indicate the median level. **** *p* < 0.001.

The stratification of measurement points based on their radial distance from the fovea is summarized in Figure 7. The control subjects had the highest IR light sensitivity along the 1° and 2° radii (r) and demonstrated a gradual decrease with a 0.8 dB difference occurring between 1° and 6°. By contrast, the AMD patients showed lower values at r = 1° compared to r = 2° by 0.3 dB. Beyond this point, the sensitivity reduction was reminiscent of our observations in the controls. The radial distribution of the MP1 results exhibits a different pattern. Normal eyes’ sensitivity was the lowest at r = 1° with a gradual increase up to 4° by 2.3 dB and a minimal (0.6 dB) decrease at 6°. A similar trend was maintained in the AMD population. However, the sensitivity values were significantly lower and demonstrated a higher IQR.

**Figure 8:**
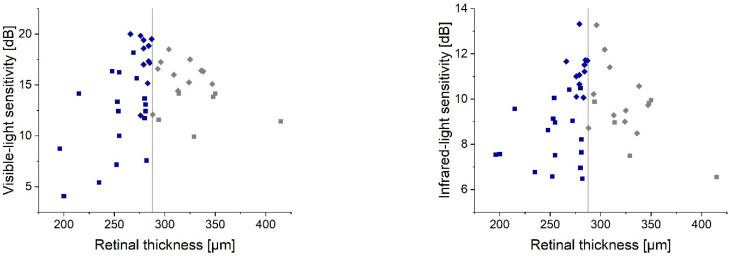
The relationship between the central subfield thickness of AMD (squares) and control (diamonds) subjects and retinal sensitivity measured using single- and two-photon approaches. The vertical line shows the median (reference) level found in the control group. The retinal thicknesses above and below the reference are marked in gray and blue, respectively.

The AMD eyes’ central subfield thickness was 272.0 (252.5; 288) µm, which was lower than that found in the controls: 287.5 (280; 312.0) µm. The median value of the control group provided a reference for the macular thickness vs. sensitivity plot (Figure 8) to create two subgroups for statistical analysis. Retinal thinning showed a significant correlation with the loss of retinal sensitivity detected with visible (ρ = 0.43, *p* = 0.02) and IR light (ρ = 0.48, *p* = 0.01). However, the increase of the central subfield thickness demonstrated a lower correlation (ρ_vis_ = 0.26 and ρ_IR_ = 0.31), which did not reach the significance level.

## 4. Discussion

We demonstrated a significant reduction in two-photon retinal sensitivity in eyes with AMD. Although we could observe a similar outcome in standard single-photon microperimetry, this method appears to be more affected by inter- and intrasubject variability.

Standard visible-light microperimetry is a popular screening option for visual-function evaluation in AMD patients beyond VA. Dinc et al. performed an analogous comparison between normal and AMD patients [13]. They recruited 30 patients diagnosed with intermediate AMD and a VA of 0.20 logMAR or better. The MP1 microperimeter used in their study had a Goldmann III size stimulus and a 1.27 cd/m^2^ background, which are standard settings also applied in our investigation, but they used a larger grid consisting of 76 retinal loci extending to 20° [13], whereas we assessed the retinal function at 44 points across 12°. They reported a mean sensitivity (±standard deviation) of 12.7 ± 2.8 dB in AMD patients and 18.0 ± 0.6 dB in age-matched controls. Although we found a similar median level in our controls, the AMD subjects were reported by Dinc and co-workers to have lower retinal sensitivity by 1.3 dB, despite being nearly 10 years younger. Their finding may indicate that any age effect may be suppressed by AMD severity, which appears to be the primary factor affecting retinal sensitivity in those patients. Still, the values reported by Dinc et al. fall within a broad IQR outlining our population, which again indicates a substantial variability in visual function between AMD patients, primarily determined by their unique course of the disease [13]. One may assume that these factors also affect two-photon retinal sensitivity.

Late-stage AMD, characterized by GA and choroidal neovascularization, has the most substantial compromising impact on retinal sensitivity. Takahashi et al. enrolled 25 patients with nonexudative AMD and GA for a comprehensive morphological and visual function examination [15]. Microperimetry was performed with the MP1 device over 57 points unevenly distributed within 10° from the center. They applied color fundus photography, SLO imaging with fundus autofluorescence, and spectral-domain OCT in order to assess the retinal structure [15]. They reported a worse BCVA (0.40 logMAR) than that of the current study and found a reduced retinal sensitivity of 8.42 ± 4.24 dB. This value decreased to 3.28 ± 2.70 dB at confined areas of photoreceptor damage and further to 1.84 ± 2.68 dB in regions with identified retinal pigment epithelium loss. Therefore, they established a clear link between retinal morphology and visual function in GA patients [15]. Munk et al. assessed patients with neovascular AMD in a longitudinal study of the effect of monthly intravitreal anti-vascular endothelial growth factor (VEGF) therapy on visual function [14]. MP1 microperimetry was performed at 14 follow-up visits scheduled within 12 months of a monthly administration of an anti-VEGF agent. A baseline VA of their 61 patients who completed that study was 0.61 ± 032 logMAR, which improved to 0.46 ± 0.36 logMAR at 12 months. Retinal sensitivity was measured within an identical angular range of 12° as applied in our investigation, but with a different (non-radial) shape of their 33-point grid pattern [14]. The mean value of 7.3 ± 4.5 dB that had been recorded before the initial injection was decreased two-fold compared to what we found in our population. A gradual improvement of retinal sensitivity was observed in the course of that study with 10.8 ± 4.5 dB at the last visit. However, between the fourth and 12th months, mean retinal sensitivity varied between 10.6 dB and 11.6 dB, which might have resulted from a higher variability of MP1. Whether the two-photon IR light stimulation, which demonstrated a narrower spread of sensitivity values, proves more advantageous in longitudinal studies requires further research.

Higher repeatability of the two-photon approach compared to a standard one-photon stimulation was demonstrated by Rumiński et al. [3] They studied a psychometric function with an optical setup featuring visible- (522.5 nm) and IR light (1045 nm) paths for single- and two-photon stimulation. Although one (IR) light source was used for both methods, visible light was a product of second-harmonic generation by a nonlinear crystal added to the visible path. Thus, only the wavelength of stimuli differed between the two conditions. Both, however, were perceived as green. Rumiński et al. reported that the psychometric function derived from IR stimuli had a two-fold (on a log scale) steeper slope than that obtained with visible light, which indicates a lower spread of values [3]. Indeed, they found that 99.7% of recorded changes fell within ±1.1 dB for two-photon testing while single-photo perception yielded ±2.2 dB. It was suggested that a higher accuracy of IR light testing results from a nonlinear behavior of the two-photon absorption process [3]. Our investigation also noted a lower spread of retinal sensitivity values in two-photon testing compared to the single-photon approach, which for the AMD and control patient was 2.1-fold and 1.7-fold, respectively. Since it was close to a two-fold difference (expected from the square ratio), this study may also support the nonlinearity of the two-photon process [3]. However, our assessment was performed on two different devices, which despite the attempts to mirror the MP1 conditions in the two-photon realm, still may not account for all existing confounders [19].

One potential explanation for increased IQR obtained with the MP1 device in the normal population is the nonuniformity of the device background. Given that our testing spanned 6° from the fixation point, one may assume that a healthy eye has an equal radial-density distribution of photoreceptors and thus retinal sensitivity. It was confirmed in the IR light testing (Figure 6). The MP1 approach demonstrated, however, a titled surface of the sensitivity map, which may suggest background intensity fluctuations over the test area. Another discrepancy between the two methods was observed in the central area. The visualization of the IR light result shows the so-called “hill of vision” with a higher sensitivity at 1° and a gradual decrease with eccentricity indicating cone-mediated vision. Although the two-photon absorption can also activate rods, cones achieve greater efficiency in this process [3,6]. Therefore, this method might be better suited for sensitivity tests performed in photopic conditions. Despite identical background illuminance, the single-photon sensitivity map showed lower values in the center than at higher angles. This might be caused by a higher contribution of rods whose density increases with eccentricity to reach a peak at approximately 20° [20]. Alternatively, radiation from the fixation circle confounded measurements at 1° despite being set at 0.5°. We used the first-generation microperimeter in our study on account of its availability at the Heidelberg Eye Clinic. We have yet to address how the latest MP3 device compares to the two-photon excitation device.

Two-photon sensitivity is a new parameter that has only recently been introduced with the first clinical outcomes reported in 2020 [4], where the age-dependency of IR light sensitivity was studied by our group in a normal population. As a proof-of-concept, five AMD subjects were also included and measured following the same protocol of 30-min dark adaptation [4]. The median scotopic sensitivity was 17.9 (17.0; 19.1) dB in young, healthy participants and 9.6 (9.4; 11.2) dB in AMD patients, yielding a difference of 8.3 dB. In the current evaluation, IR light sensitivity was reduced only by 2.4 dB in the diseased eyes. The reason for a smaller effect might be an insignificant age difference observed here, while in the earlier study, the median value of the healthy population was 44.1 (31.6; 53.1) years. Another explanation is faster degradation and loss of rods than cones in AMD [21,22], which may result in the compromised visual function of dark-adapted eyes. Therefore, performing dark-adapted microperimetry with two-photon stimuli may prove a more sensitive measure of disease progression and its functional effect. This conjecture, however, should be validated in a clinical study.

The assessment of retinal morphology in AMD patients has improved our knowledge about the pathomechanism of this disease [9,10]. A routine application of OCT in AMD patient management has led to the introduction of various retinal biomarkers, which can predict the disease’s progression and a patient’s functional outcome [23]. Central retinal thickness is one parameter that is readily accessible to clinicians as it is automatically generated in each OCT macular report. Although OCT devices may differ in anatomical landmarks used for thickness measurements, a clear correlation has been observed between retinal thickness changes and eye sensitivity [24,25]. Alexander et al. assessed 14 neovascular AMD patients during anti-VEGF therapy [24]. They studied the association between the OCT-derived central retinal thickness and microperimetry results, showing a coefficient of determination (R^2^) of 0.69 for the nonlinear fit of their data clusters. Alexander et al. also demonstrated that an excessive increase or decrease in retinal thickness results in degradation of the visual function [24], which is consistent with the findings of our analysis. A potential explanation is that retinal thinning may indicate atrophy, while active neovascularization may increase retinal thickness [24]. Both conditions yield a significant reduction of retinal sensitivity. However, predicting visual function by measuring retinal thickness would appear to have limited application given the broad range of median sensitivity points along a vertical line demarcating the reference level. The Spearman’s rank correlation coefficient also indicates a worse prediction value in cases with increased retinal thickness, perhaps because only a few study cases presented subretinal fluid. However, in the report by Sabour-Pickett et al., a significant correlation was noted with a Pearson correlation coefficient of −0.59 for their entire cohort of patients treated with anti-VEFG intravitreal injections due to retinal fluid build-up or cyst formation [25]. Thus, in a more uniformly phenotyped population, the predicting value of the retinal thickness parameter may increase. Interestingly, despite differences in the mediation of two- and single-photon vision, both instruments appear to have a comparable predictive value.

## 5. Conclusions

AMD reduces retinal sensitivity to IR light. We also observed this in standard visible-light microperimetry. Although the two-photon device demonstrated a lower data spread, we have yet to elucidate whether this arises from the nonlinearity of the two-photon absorption process or is due to the limitations of first-generation visible-light microperimetry. In addition, we found a significant correlation between retinal sensitivity parameters and the reduction of the central subfield thickness. However, further studies are necessary if we are to link this anatomical change and other retinal and choroidal biomarkers with two-photon vision and obtain a better understanding of the clinical factors affecting this new functional parameter.

## Data Availability

All available data generated or analyzed during this study are included in this published article.
